# Supplementary material for: Professional soccer practitioners’ perceptions of using performance analysis technology to monitor technical and tactical player characteristics within an academy environment: A category 1 club case study
Source: PLoS One. 2024 Mar 7;19(3):e0298346. doi: 10.1371/journal.pone.0298346 (PMC10919864; doi:10.1371/journal.pone.0298346)
Supplement: Attachment — Submitted filename: Plos One. Review.pdf [file pone.0298346.s001.pdf]

**Plos One**

**Professional soccer practitioners' perceptions of using performance analysis technology to monitor technical and tactical player characteristics within an academy environment**

Thank you for the opportunity to review this relevant manuscript. I welcome studies that introduce the professional soccer practitioners' perceptions of the application of performance analysis technology within an academy environment and try to elucidate the perceived importance that professional soccer club practitioners place on monitoring technical and tactical player characteristics, current performance analysis practices, and barriers to implementing wearable technology. In fact, I am open to be persuaded to deep understand this relevant topic.

Hence, I am some sympathy with the author's intentions. In addition, the authors provide a decent description of the introduction, process, and some proposals for future research. The topic represents contemporary interest, and the scope of the work and is appropriate for *Plos One.* I think it is very important to conduct studies like this one, because provide different key points to bring to light studies whose aim is to review what has been published previously on this topic in a rigorous way. This study contribute to the understanding the importance that professional soccer club practitioners place on monitoring technical and tactical player characteristics and the barriers to implementing wearable technology to aid with monitoring within an academy setting. In fact, this study is particularly important because, as the authors indicate, the publications on this topic have a special gap, so it is necessary to group all these papers to have a better understanding of the results obtained previously.

From my point of view, the main strength of the manuscript is the methodology and the results presented clearly (qualitative and quantitative). Apart from this general commentary about the review, more details of some parts of the manuscript (strengths, weakness and questions) are found hereafter.

Abstract.

1. For future study should be indicated at the end of the abstract as well as in the conclusions section some directions about the results in relation to the work

Introduction

2. It is well written and structured. It is a good starting point to place the reader. However, truly think that is very short and need more information and important literature.
3. The introduction doesn't explain the basic concepts used in the paper neither qualitative nor quantitative similar analysis. Some of these are presented in the methodology section, in terms of how they were measured, however it would have been necessary to clarify their general content in the introduction section.
4. The last part of the introduction (5º last paragraphs since "Implementing technology ….") are confused and hard to understand.

Methodology

5. **Part A.** To rewrite the section participants.
6. Table 1. Add information about the variables (measures of each value) methodology clearly explained and justified.
7. Is necessary describe the sample more deeply. Exclusion and inclusion criteria
8. Please, add information about the sample size calculation with G-Power or another software.
9. A schematic representation is necessary to elucidate the study.
10. Part B. Add the same information solicited in Part A
11. Statistical analysis. (What software was used to Part A and for Part B?)
12. Add information about values of effect sizes of Pearson's chi-squared

<u>Results.</u>

13. I suggest the possibility to create a logistic regression, because is used to predict the outcome of a categorical variable based on the independent or predictor variables. Is possible to create in part A?
14. Please add information about effect size, maybe V-Cramer or othr. In fact, when you have information about effect size you can discuss more precise the discussion.
15. Truly, I think that the figure 1 and figure 2 is not necessary if the information is added in the text. The same with figure 4 and 5.
16. In Part-B interviews. I need that you elucidate the information about categories and their categorizations and also what coding of data collections was used? I understand that you use software similar to WEFT QDA, NVIVO, QUIRKOS?
17. I think that you need reduce the sentences of each question.
18.

Discussion

19. Discussions is appropriate, making reference to the results of other studies, but, the theoretical and practical implications of the research are vaguely mentioned. It necessary improve it.

Conclusions

20. The conclusions respond to the objectives of the study but prospective should be included.
